# A novel keratin 13 variant in a four‐generation family with white sponge nevus

**DOI:** 10.1002/ccr3.1073

**Published:** 2017-07-29

**Authors:** Stephanie B. de Haseth, Egbert Bakker, Maarten H. Vermeer, Hakima el Idrissi, Tjalling Bosse, Vincent T.H.B.M. Smit, Anna Terron‐Kwiatkowski, W.H. Irwin McLean, Alexander A.W. Peters, Frederik J. Hes

**Affiliations:** ^1^ Department of Gynaecology Leiden University Medical Center Leiden The Netherlands; ^2^ Department of Clinical Genetics Leiden University Medical Center Leiden The Netherlands; ^3^ Department of Dermatology Leiden University Medical Center Leiden The Netherlands; ^4^ Department of Pathology Leiden University Medical Center Leiden The Netherlands; ^5^ East of Scotland Genetics Service Ninewells Hospital Dundee UK; ^6^ Centre for Dermatology and Genetic Medicine University of Dundee Dundee UK

**Keywords:** De novo variant, *KRT13*, mucosal neoplasia, White sponge nevus

## Abstract

We report a novel KRT13 germ line variant that causes white sponge nevus (WSN) with mucosal dysplasia. Genital, vaginal, and cervical WSN were observed in four female patients, of whom two had premalignant cervical lesions at young age. Two of the 12 patients with oral WSN developed oral squamous cell carcinoma.

## Introduction

White sponge nevus (WSN) is a rare, benign, and autosomal dominant disorder, with no sex predilection. The disorder was first reported by Hyde in 1909, 1 and the term “white sponge nevus” was coined in 1935 by Cannon 2. WSN (OMIM 193900) is caused by germ line variants of the keratin genes *KRT4* or *KRT13*, located, respectively, at chromosomes 12q13 and 17q21‐q22 3, 4.

White sponge nevus is characterized by white spongy plaques of the mucosa and predominantly affects nonkeratinizing stratified epithelia such as the oral mucosa. Extra‐oral lesions are found in other mucosal sites, but are relatively uncommon in the absence of oral manifestations. Extra‐oral mucosal sites include the genital, laryngeal, and esophageal mucosa 5, 6, 7, 8, 9. Extra‐oral affected sites have been shown to arise only from *KRT13* variants. The most frequent site affected was the vaginal mucosa 10. The histological features of WSN are acanthosis, parakeratosis, cytoplasmic clearing of spinous layer keratinocytes, and characteristic perinuclear eosinophilic condensations that correspond to abnormal aggregation of cytokeratin tonofilaments 11. The findings through microscopic examination demonstrates that cytology can be very useful in distinguishing WSN from other histopathologically similar lesions 8.

Clinically, the lesions are characterized by the presence of soft, white, and spongy plaques in the mucosa, of variable sizes. The gynecological abnormalities are usually asymptomatic, although pruritus, burning, and pain have been reported to follow irritating stimuli 12. The differential diagnosis for vulvar leukoplakia is vast and includes inflammatory, premalignant/malignant, infectious, and congenital abnormalities. Oral WSN may be confused with leukoplakia, candidiasis, pachyonychia congenita, hereditary benign intraepithelial dyskeratosis, Darier's disease, dyskeratosis congenita, lichen planus, lupus erythematosus, chemical burns induced by tobacco or betel nut use, and syphilis 8. Distinction in this differential diagnosis is essential in terms of differences in treatment and prognosis.

The majority of WSN are described in case reports, and only a few larger families with WSN have been reported and of these, only two had a *KRT13* gene defect 4, 8, 13. Here, we report a four‐generation family with oral and gynecological manifestations of WSN caused by a novel germ line variant in the *KRT13* gene.

## Subjects and Methods

### Molecular genetic analysis

The index patient was sequenced for germ line variants in the *KRT4* and *KRT13* genes. DNA was obtained from peripheral blood by standard methods. Analysis of KRT4 and KRT13 sequences was performed by PCR amplification of the coding regions and splice sites, including some intronic sequences, with flanking‐specific primers (primers and conditions available upon request) followed by standard Sanger sequencing. Sequence analysis using Sequence Pilot by JSI Medical Systems Corp.(USA) based on KRT4 (NM_00272.2) and KRT13 (NM_153490.2).

Characterization of the detected variants at mRNA level was performed by use of in silico prediction programs and by analysis of the transcript by use of RT‐PCR on total RNA isolated from a short‐term lymphocyte culture 14, 15.

### Clinical analysis of family members

A family gathering was organized for obtaining written informed consent and DNA testing. The procedure was approved by the medical ethical committee of the LUMC (P12.113). A total of 20 family members donated blood for DNA testing, and 22 persons completed a questionnaire on manifestations of WSN, including age at diagnosis and anatomical location. In addition, we asked them whether they had complaints of the disorder, whether their children were affected, and whether they had any tumors or any other important diseases. Moreover, women were asked whether they participated in the national cervical cancer screening service. All family members underwent physical examination by a dermatologist (M.V.). Seven of the 12 women opted for an examination by a gynecologist (A.P.). The gynecological examination included an inspection of the genital area and a pap smear.

### Immunohistochemistry

Formalin‐fixed paraffin‐embedded tissue blocks were sectioned at a thickness of 4 *μ*m, dried, dewaxed, and rehydrated by 0.3% H_2_O_2_/methanol. Antigen retrieval was achieved by microwave oven treatment in 10 mmol/L citrate buffer, ph 6.0, for staining of p53. Sections were incubated overnight with monoclonal TP53 (clone DO‐7; Neomarkers, Ab‐5) and 1:800 Keratin13 (clone AE8; Life Span Biosciences). The sections were then incubated for 30 min with a secondary antibody (Poly‐HRP‐GAM/R/R; DPV0110HPRP; Immunologic). Diaminobenzidine tetrahydrochloride was used as a chromogen. The slides were counterstained with hematoxylin.

### Loss of heterozygosity analysis

Loss of Heterozygosity (LOH) analysis was performed as previously described 16. In short, 14 microsatellite markers distributed on 12 chromosome arms, including two markers on chromosome #17, were amplified by DNA PCR using fluorochrome‐labeled primers. The PCR products were evaluated on a capillary sequencer (ABI). The fluorescence intensity ratio of the two alleles of the tumor DNA isolated from the two tumor samples and were normalized using normal DNA from the same patient. A ratio ≤0.75 was interpreted as AI (Allelic Imbalance) and a ratio ≤0.5 as LOH.

## Results

### Clinical details

**Figure 1:**
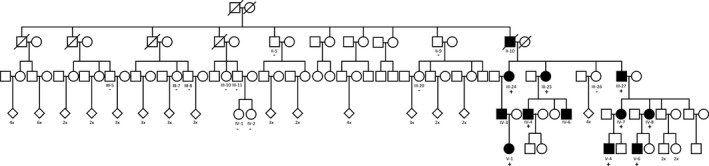
Pedigree of the family. Family members clinically affected with WSN are shown in black. White symbols represent unaffected family members. The presence (+) and absence (−) of the variant in tested individuals are shown under the symbols, that is, circle (woman) and block (man). Diamond symbols represent either man or woman, with the number of family members annotated under the diamond. A diagonal line represents that the person is deceased.

The index patient (IV‐7, arrow in Fig. 1) was diagnosed with WSN when she visited the Gynaecology Department for anticonception at an age of 18 years. In 1991, microscopic analysis of the cervix cytology showed Papanicolaou score Pap3A (mild dysplasia). Upon review in retrospect, the morphology was also compatible with WSN, except that perinuclear eosinophilic condensations were not visible. Additional testing on this biopsy revealed no HPV involvement. At yearly follow‐up, she continuously had a cytological diagnosis of Pap2 or Pap1. Repeat histology showed hyperplastic cervical squamous epithelium and mild inflammation, but no dysplasia. She did not have any complaints of dyspareunia or postcoital bleeding. Last colposcopic examination showed thick white epithelium covering most of the vagina and the ectocervix. Exploration of oral cavity revealed white, spongy, rough‐looking lesions extending over the whole oral mucosa, floor of the mouth, and side of the tongue, which could not be removed by scratching.

The sister of the index patient (IV‐8) was subsequently diagnosed with WSN. At the age of 16 years, cervical smear showed a Pap2 diagnosis. On last gynecological examination, she was very sensitive in the genital area and with mucosa prone to bleeding. Patient III‐25 had a Pap3A in her cervical smear at an age of 27 years. In these three women (IV‐7, IV‐8, and III‐25), cytoplasmatic aggregates of keratin were not reported, but a “HPV‐cytopathic effect” (with possible viral infection), pseudokoilocytosis, and endocervical cylindric and squamous metaplastic cells. Subsequently, HPV testing was performed on the three Pap smears and was negative, despite the cytology with viral‐like features. Patient III‐24 did not give permission to study her historical medical files. Gynecological WSN was observed in all four adult female patients. A minor female (V‐1) with oral WSN was not examined by a gynecologist.

**Figure 2:**
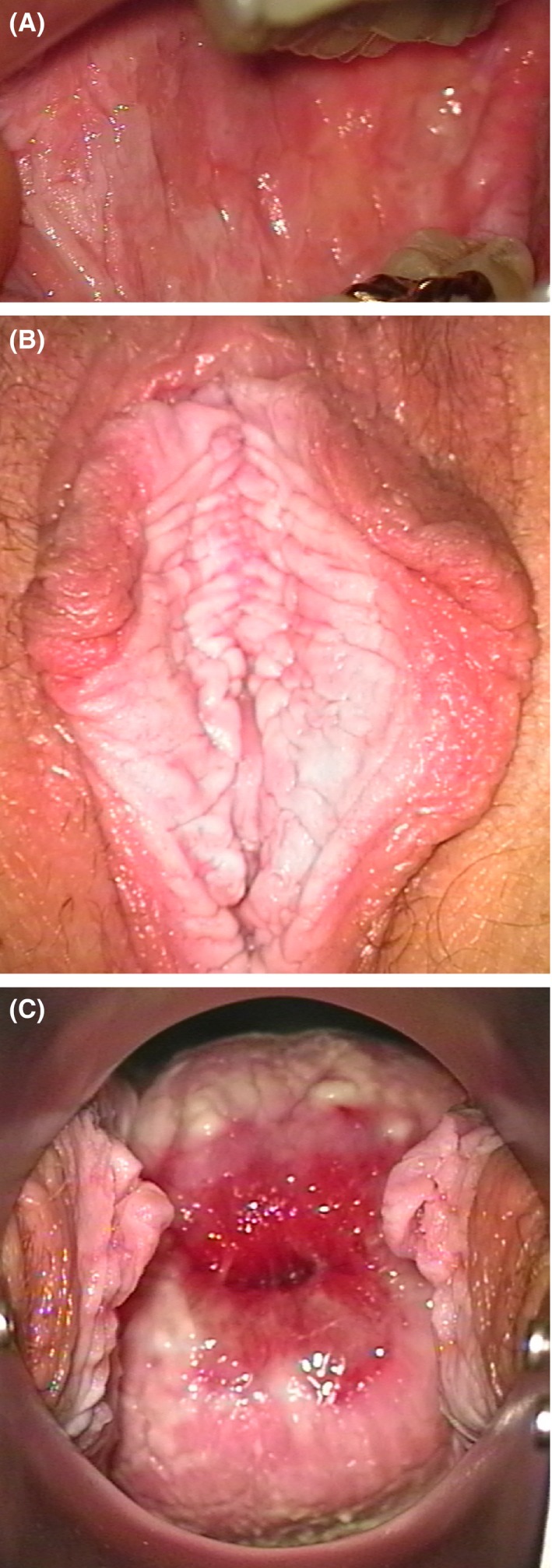
Oral WSN (A): white to light rose‐colored, fissured plaques of the buccal mucosa and ventral surface of the tongue of one of the family members (not specified, because of privacy reasons). Genital WSN: thick white epithelium lines the inner surface of the labia minora (B) and extends over the introitus (C). Family member not specified, because of privacy reasons.

All variant carriers (see below) had oral WSN on inspection. Oral biopsies were taken from patients III‐25, III‐27, IV‐3, and IV‐4, and all showed a histology with parakeratosis and mucosal hyperplasia resembling WSN. The grandfather (II‐10) and his daughter (III‐24) both had an oral tumor, respectively, aged 77 and 65 years. Histopathological analysis showed well‐differentiated squamous cell carcinomas deriving from the oral mucosa. Adjacent mucosa showed no WSN and HPV testing was negative. Examples of clinical manifestations within this family are depicted in Figure 2. None of the examined males displayed genital WSN.

### Molecular genetic analysis

Firstly, a heterozygous amino acid substitution in exon 2 of *KRT4* (c.845G>A, p.Arg282His) was identified in the index patient. However, this variant was not present in her affected sister (IV‐8). As the two sisters are similarly affected, this variant did not appear to cause the phenotype. Moreover, this *KRT4* variant is located outside the conserved keratin domains where the vast majority of pathogenic variants are located (www.interfil.org).

**Figure 3:**
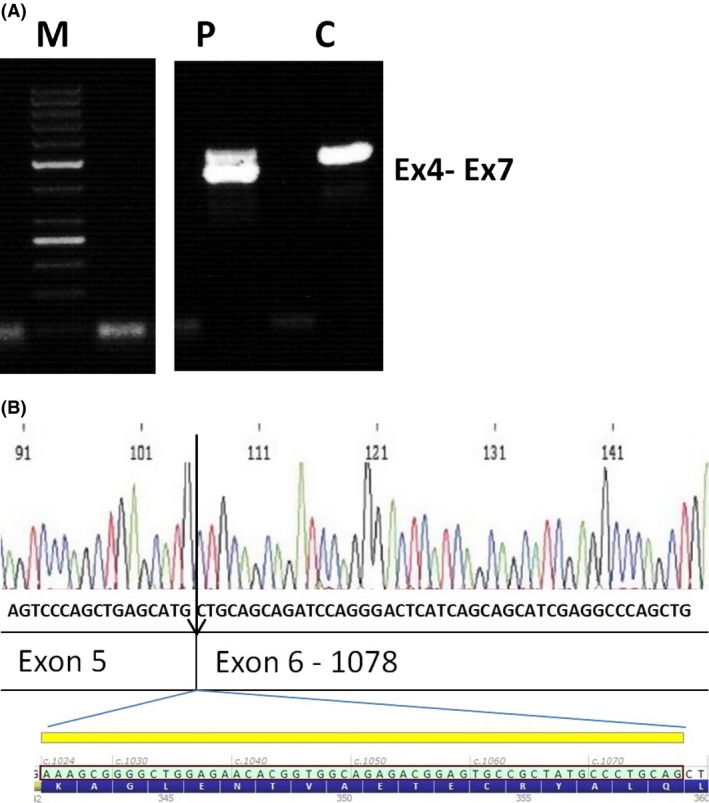
cDNA PCR of the region exon 4‐7 (A) shows an aberrant (shorter) fragment in the patient versus a control sample. Sequence analysis of aberrant band (B) shows a deletion of the first part of exon 6 (1024–1077 = 54 base pair).

Secondly, both sisters were found to carry a heterozygous 32‐base pair deletion in intron 5 of *KRT13* (c.1023+23_1024‐39del). In silico analysis of the intronic deletion by use of several splice prediction programs, visualized by the Software program Alamut (Interactive Biosoftware, France), did not show any predicted effect on the splicing. The deletion did however take three possible branch points. In vitro RNA analysis by use of RT‐PCR initially showed two very weak cDNA bands, indicating aberrant splicing of exon 6. By PCR using primers in exon 4 and exon 7, the aberrant cDNA fragment was clearly detectable in the RNA of the patient (III‐25) Figure 3A.

Sequence analysis of the cDNA shows a heterozygous deletion of 54 bases from the mRNA (r.1023‐1077) Figure 3B, which causes an in‐frame loss of 18 amino acids from the KRT13 protein (p.Lys342_Gln359del).

Subsequently, we tested as many family members as possible for cosegregation analysis of the *KRT13* defect. We found that the variant was present in all nine affected family members that were available for testing but was absent in 11 unaffected family members where DNA was available (Fig. 1).

**Figure 4:**
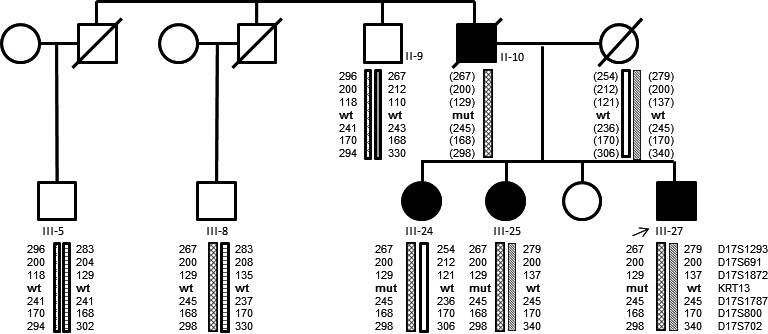
Haplotype analysis by use of highly informative STR loci flanking the KRT13 loci within 5 cM of the gene. The affected haplotype (hatched pattern) as seen in the index patient III‐27 is also detected in an unaffected relative, individual III‐8.

Haplotype analysis by use of six highly polymorphic STR markers flanking the KRT13 gene shows within this large Dutch family that this germ line variant did occur de novo in the affected (WSN and oral squamous cell carcinoma) great‐grandfathers DNA and segregates on a haplotype which does not carry the defect in the unaffected part of the pedigree (Fig. 4).

**Figure 5:**
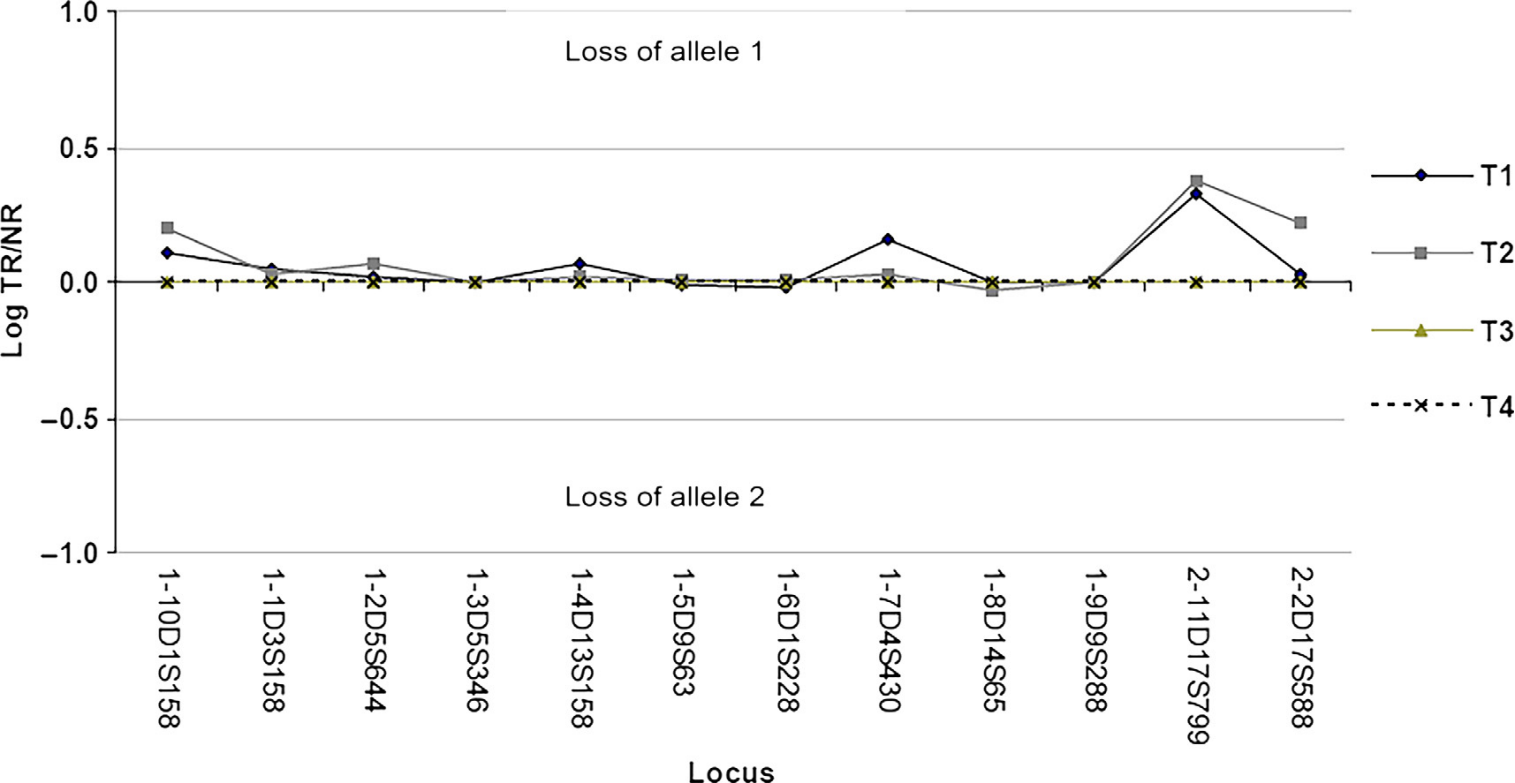
LOH analysis. Tumor 1 (T1): squamous cell carcinoma of right inferior jaw, squamous cell carcinoma of right tonsil (T2), control tumor (T3), and normal tissue (T4).

Genetic analysis on the squamous cell carcinoma of patient II‐10 with markers on chromosome 17 (D17S799 and D17S588) demonstrated LOH at chromosomal location 17p11.1 (where the p53 tumor suppressor gene is located) but not on 17q21 where the *KRT13* gene is located (Fig. 5). Immunohistochemistry showed a wild‐type staining pattern of P53 in the squamous cell carcinoma.

Immunohistochemical staining with keratin 13 antibodies showed normal epithelial expression in the cervical tissue of the index patient. In the oral squamous cell carcinoma of patient II‐10, a lower intensity of the keratine 13 expression was observed.

## Discussion

We report a large WSN family of four generations with 12 affected family members. Most commonly, keratin disorders such as WSN are caused by missense or small in‐frame insertion/deletion variants clustered in the helix 1A domain of the keratin polypeptide. Here, we identified a more downstream variant – a 32‐base pair deletion in intron 5 of *KRT13* (c.1023+23_1024‐39del). We demonstrated (Fig. 3) that this intronic deletion has an effect on the splicing by quantitative RT‐PCR and predicts an in‐frame deletion of the protein. So far, only missense variants in exon 1 of human keratin 13 have been shown to be associated with WSN 4, 13, 17, 18, 19. Single amino acid alterations have shown to produce defective keratin intermediate filaments which form dense cytoplasmic aggregates in upper spinous layers 4. Apparently, larger defects in the structure of *KRT13* as identified here also result in a WSN phenotype.

The variant showed complete cosegregation with the WSN phenotype in this family. Of the three large families reported so far, two also showed full penetrance of the disease 4, 13. In one family, without molecular genetic analysis of the keratin genes to confirm the diagnosis, incomplete penetrance of the WSN phenotype was reported 8. The absence of both the *KRT13* variant as well as any clinical phenotype in the left side of the pedigree supported by haplotype analysis prove that the variant occurred de novo in the grandfather of the index patient. To our knowledge a de novo variant – also in *KRT13* – has been described once in WSN, but this was based only on clinical information 18.

Our family presents a strong genotype–phenotype correlation, that is, all nine carriers of the *KRT13* germ line variant are affected with WSN. Remarkably, all our patients reported that they were affected from an early age and that oral WSN was already recognized from birth. Moreover, we observed genital, including vaginal, and cervical WSN lesions in all four adult women. The index patient, her sister, and paternal aunt had abnormal cervical smears at a relatively early age (18, 16, and 27 years, respectively) in the absence of any apparent risk factors as sexarche, or more than one sexual partner. WSN presenting as genital lesions has been described; however, this was in the absence of vaginal or cervical involvement 7.

Remarkably, the grandfather of the index patient and also his daughter developed a mucosal cancer. Marker analysis on chromosomal location 17q21, in his oral squamous cell carcinoma (patient II‐10), did not show LOH, suggesting that *KRT13* does not act as a tumor suppressor gene. Immunohistochemical staining with keratin 13 antibodies showed strong expression in cervical epithelial cells. The oral squamous cell carcinoma also showed positive staining, although with somewhat lower intensity. While a study of an oral squamous cell carcinoma showed significant downregulation of keratin 4 and keratin 13 expressions 20. Keratin disorders are usually caused by missense variants that result in aggregates of keratins. As we did not observe the characteristic perinuclear condensations of keratin aggregates in the cervical and oral tissues of the individuals analyzed with a non‐missense variant, it is tempting to speculate that there is a genotype–phenotype correlation in keratin 13 defects. Whether this or other keratin disorders have (pre)malignant potential remains to be elucidated. To our knowledge, these are the first findings of (pre)malignancy in WSN. This observation warrants further investigations in other WSN families and also to evaluate whether these manifestations are related to a specific type of *KRT13* germ line variant.

The diagnosis of WSN should be made on detailed patient and family history, inspection of mucosal lesions by an experienced dermatologist, histopathology, and molecular genetic analysis of the *KRT4* and *KRT13*. The cytological abnormalities observed and described by several independent cytologists was not the result of HPV infection, as HPV testing was negative. Clearly, the cytology of genital WSN mimics that of HPV‐related changes. Given the wide differential diagnosis of white mucosal lesions, an accurate diagnosis is important for treatment and counseling of patients. WSN are reported to respond on treatment with tetracycline, penicillin, or chlorhexidine 21, 22, 23. However, in cases where there is a lack of symptomatic disease, conservative treatment is a good alternative 8. So far, guidelines for clinical surveillance for WSN are not available. Because of the uncertainty of the tumorigenic potential of the disorder, our patients were given the opportunity of an annual routine gynecological checkup. Alternatively, patients with WSN can follow national prevention programs. In the Netherlands, women between the age of 30 and 60 years are offered screening every 5 years with analysis of cervical smears. In addition, it seems prudent to instruct patients to consult a dermatologist with any suspected mucosal lesion.

In addition, a correct diagnosis is not only important for providing appropriate management and surveillance but also for counseling of family members in the case of WSN. Two of our family members reported to have white oral lesions. However, examination by the dermatologist showed candidiasis and neither of these individuals carried the *KRT13* variant. Both could be reassured that they did not have WSN and could not pass this disorder on to their offspring. Therefore, DNA analysis could be offered to family members, mainly to rule out the diagnosis of WSN. Considering recommendations for the surveillance of germ line variant carriers of a keratin defect, more studies on the clinical manifestations of WSN are warranted.

## Conclusion

We describe a novel *KRT13* variant, with a de novo origin in the great‐grandfather, in a four‐generation family that causes WSN with full penetrance. This report adds a number of novel aspects to what is known about WSN. First, other mutations beside specific missense variants in the 1A domain of the keratin 13 polypeptide can cause WSN. Second, WSN involvement can extend to vagina and cervix. Third, there is a remarkable incidence of premalignant cervical lesions in affected women and we also report the first malignant oral mucosal lesions in WSN.

## Conflict of interest

The authors declare no conflict of interest.

## Authorship

SBH: performed data analysis and writing. EB, HI, TB, VTHBMS, and ATK: performed experimental work and writing. MHV, AWP: performed patient examination, obtained clinical data, and performed writing. WHIM, AAWP, and FJH: performed supervision, contributed to study concept and design, and performed critical revision of the manuscript.
